# Validation of the VitaBit Sit–Stand Tracker: Detecting Sitting, Standing, and Activity Patterns

**DOI:** 10.3390/s18030877

**Published:** 2018-03-15

**Authors:** Nathalie M. Berninger, Gill A. ten Hoor, Guy Plasqui

**Affiliations:** 1 Department of Work and Social Psychology, Faculty of Psychology and Neuroscience, Maastricht University, P.O. Box 616, 6200 MD Maastricht, The Netherlands; gill.tenhoor@maastrichtuniversity.nl; 2 Department of Human Biology, Maastricht University, P.O. Box 616, 6200 MD Maastricht, The Netherlands; g.plasqui@maastrichtuniversity.nl

**Keywords:** sedentary behavior, VitaBit, accelerometer, validation, sensitivity, specificity, positive predictive rate, negative predictive rate

## Abstract

Sedentary behavior (SB) has detrimental consequences and cannot be compensated for through moderate-to-vigorous physical activity (PA). In order to understand and mitigate SB, tools for measuring and monitoring SB are essential. While current direct-to-customer wearables focus on PA, the VitaBit validated in this study was developed to focus on SB. It was tested in a laboratory and in a free-living condition, comparing it to direct observation and to a current best-practice device, the ActiGraph, on a minute-by-minute basis. In the laboratory, the VitaBit yielded specificity and negative predictive rates (NPR) of above 91.2% for sitting and standing, while sensitivity and precision ranged from 74.6% to 85.7%. For walking, all performance values exceeded 97.3%. In the free-living condition, the device revealed performance of over 72.6% for sitting with the ActiGraph as criterion. While sensitivity and precision for standing and walking ranged from 48.2% to 68.7%, specificity and NPR exceeded 83.9%. According to the laboratory findings, high performance for sitting, standing, and walking makes the VitaBit eligible for SB monitoring. As the results are not transferrable to daily life activities, a direct observation study in a free-living setting is recommended.

## 1. Introduction

Consequences of uninterrupted sitting entail high risks of developing metabolic and cardiovascular diseases, certain types of cancers, and all-cause mortality [1,2]. Additionally, first evidence suggests that negative psychological impact caused by sedentary behavior (SB) should not be neglected [3,4]. The psychological and physical consequences of SB were found to occur independently of other health-related behaviors, such as leisure time moderate-to-vigorous physical activity (PA) [5,6]. Although high levels of PA, i.e., more than 5 h of jogging per week, might mitigate the risk of overall sitting time, it was found that it does not eliminate the risks associated with TV watching time of more than 5 h [7]. Therefore, it is likely that PA cannot mitigate the risks of specifically uninterrupted overall sitting time. Given the rising estimated prevalence of SB, increased attention is drawn to interventions that aim to overcome detrimental sitting and subsequent increased risk [8,9].

When developing and evaluating these SB interventions, objective measurement and monitoring tools for SB and its antagonist behaviors (standing or walking) are indispensable. In order to create awareness for a putative behavioral change, those measurements need to display deviations of the users’ actual sit–stand–walk patterns from the recommendations [9,10]. Furthermore, they are needed to refine recommendations for activity patterns (e.g., “It is recommended to interrupt sitting at least once per hour by either 10 min standing or 2 min walking”) and to develop and improve behavioral change programs [6]. Yet, a valid direct-to-consumer SB monitor is currently not available [11]. 

Current objective best-practice trackers applied in SB research with high accuracy are tri-axial accelerometers like the ActiGraph (GT3X+, ActiGraph, Pensacola, FL, USA) or posture monitors like the ActivPal (PAL Technologies Ltd., Glasgow, UK) [11]. However, these still come with high-cost soft- and hardware or require profound knowledge on data analysis [12]. Current direct-to-consumer monitors (e.g., Flex 2, Fitbit) focus more on PA than on posture detection such as sitting and standing [13,14,15]. The VitaBit device used for the current study distinguishes sitting, standing, and walking and offers, among others, a monitoring tool for the end user. It further provides behavioral change specialists with a tool to interact (e.g., e-mails or push-messages) and individually adapt their health suggestions (e.g., based on activity data, compliance, or goal-setting behavior) to the end-user. The standard list price on the website is €99.95 (excluding VAT), including the monitor and one-year usage of apps, web portal, and data analysis/export functionality, while pricing for over 10 monitors is upon request.

In this paper, we examine the validity of the new VitaBit (VitaBit Software International B.V., Eindhoven, The Netherlands) accelerometer in a laboratory and free-living setting with direct observation and ActiGraph output as criterion measurements, respectively. Regarding device performance measurements, we were specifically interested in the evaluation of binary classifiers, more detailed in sensitivity, specificity, positive (PPR, also referred to as precision) and negative (NPR) predictive rate. 

## 2. Materials and Methods

### 2.1. Sample

Fourteen participants (11 females, three males) volunteered for the laboratory study (nine females, two males) and/or the free-living study (six females, one male) and its sub-study (six females, three males). Volunteers were required to have an Android or iOS smartphone and to be willing to download the VitaBit smartphone application (https://www.vitabit.software/en-GB/), aged between 18 and 50 years, and fluent in English or German. People with any condition preventing them from performing the exercise protocols (Medical Screening Questionnaire in Appendix A) were excluded from the study. Prior to participation, informed consent was obtained. Ethical approval had been obtained by the Ethics Review Committee Psychology and Neuroscience, Maastricht University, the Netherlands (ECP-04-09-12). The cleaned datasets and the code for analyses in R can be found in DataverseNL (http://hdl.handle.net/10411/H5PZCT).

### 2.2. VitaBit as a Measurement Tool

The VitaBit device is a small, cuboid accelerometer (3.9 × 1.4 × 0.85 cm, 4.8 g) with long battery life (>30 days with auto-synchronization) that is worn at the thigh. It can be placed in the front pocket of the pants or be attached to tights or pocket-less trousers and skirts, as a magnetic clip helps to fix it to a garment layer. The VitaBit hardware includes a Micro-Electro-Mechanical Systems (MEMS) motion sensor, which is a tri-axial linear accelerometer and a wireless microcontroller targeting, among others, Bluetooth applications. The sensor is capable of detecting accelerations with an amplitude range of −16 to +16 g and 6D/4D orientations with a sampling rate of 33 Hz and an output data rate of 30 s. Via a proprietary algorithm, the processor samples pedometer data to calculate whether the output for a 30-s period is categorized as walking; if the output is not walking, the algorithm differentiates between sitting and standing. Thereby, the VitaBit regulatory recalibrates, eliminating the necessity of a determined device orientation in space, which makes it easy to deploy on a daily basis. The device stores activity and sitting data for at least 30 days, which can be synchronized with a connected smartphone application (requiring iOS 7.1/Android 4.3 or higher) via a Bluetooth Low Energy connection. After synchronization of the device with the smartphone app, the app sends the data via a wireless Internet connection to a back-end server. The data are processed nearly in real time and securely stored in a time series database, before they are used by a web-based analytics portal (https://www.vitabit.software/en-GB/).

### 2.3. Protocol

**Figure 1:**
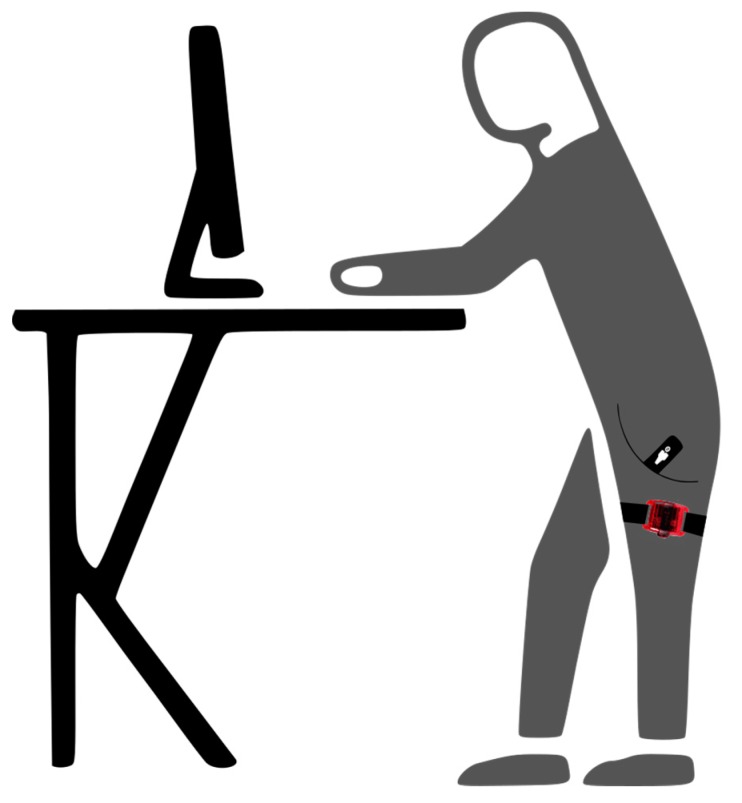
Illustration of wearing locations when wearing both devices simultaneously.

This observational study consisted of two parts, a laboratory part in which we examined the validity of the SB tracker in a controlled and directly observed laboratory setting, and a free-living part, in which the VitaBit was validated against one of the current best-practice trackers, the ActiGraph [11]. During the three parts of the lab-controlled study, participants wore the VitaBit in their pockets or attached the monitor close to their pocket using a magnet in case no or only loose pockets were available. Pre-inspection of the data revealed no difference between the two ways of attachment. They were instructed to walk, sit down, and stand up in different predetermined paces while being observed by the experimenter (see Section 2.3.1). Within the free-living part, a “sub-study” was conducted to validate the ActiGraph’s eligibility as a measurement criterion. A sub-sample of subjects wore the ActiGraph on their thigh using an elastic band during the laboratory conditions (Figure 1). Volunteers participating in the free-living part of the study wore both devices simultaneously on their thigh for at least one typical week- or weekend day. 

After the health screening and informed consent were obtained, participants were shown how to install and subscribe to the smartphone app before they were given the VitaBit device.

#### 2.3.1. Validity of the VitaBit in a Controlled Setting

The lab protocol consisted of three different parts. In the first part (see Section 2.3.1, laboratory part 1), the focus was on the distinction between the three activity postures: sitting, standing, and pre-determined paces of walking indicated by a digital metronome. Transition and acceleration periods between the posture changes were excluded from the analyses. The second part (see Section 2.3.1, laboratory part 2) concentrated on performance values with (laboratory part 2a) and without (laboratory part 2b) transitions between posture changes (e.g., sitting to standing; walking to sitting) and on somewhat natural, individual activity paces. A transition interval was defined as the 30 s before and after posture changes, starting or ending with a transition of maximum 5 s. Since the third part (see Section 2.3.1, laboratory part 3) was dedicated to the accelerations of sitting down and getting up, participants performed those transitions in three different paces. The sitting and standing periods following those transitions were analyzed.

Starting times of the three laboratory parts were noted by the researcher. The time remaining before the beginning of an upcoming activity was read aloud and counted down. After following the observation protocol, volunteers were to synchronize the VitaBit sensor with their VitaBit application on their phones.

##### Laboratory Part 1—Distinction between Sitting, Standing, and Walking

**Figure 2:**
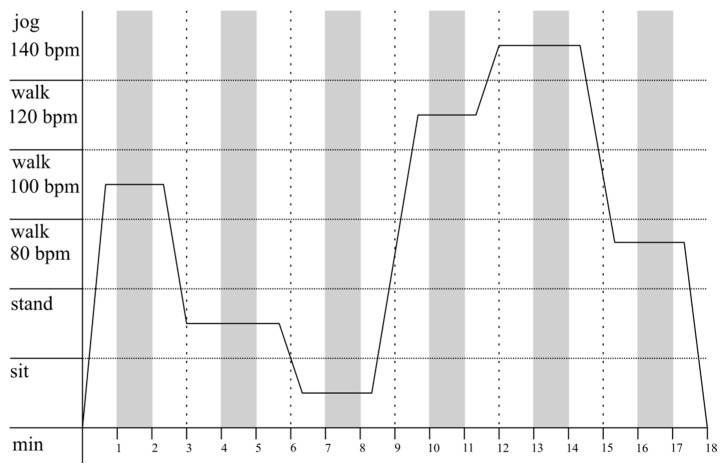
Laboratory part 1: Example protocol of activity distribution as a function of time. Every activity is randomly allocated to one of the six periods of 3 min. While white areas are the time slots in which participants performed transitions; gray areas depict the windows of analysis.

In laboratory part 1, participants followed a randomized protocol of the three postures: sitting, standing, and standardized paces of walking activity (walking 80 beats per minute (bpm), 100 bpm, 120 bpm, jogging 140 bpm). Every activity was allocated to one of the six periods of 3 min each, where the first and the last minute served as transition or recovery periods and the minute in the middle of the interval was analyzed. Laboratory part 1 yielded 6 min of data per participant (Figure 2).

##### Laboratory Part 2—Influence of Transitions and Natural Paces on Validity

**Figure 3:**
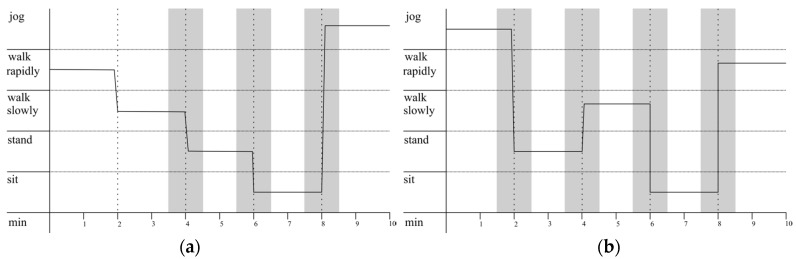
Laboratory part 2: Examples of two different possible activity protocols as a function of time. Thirty-second periods before and after posture changes within the laboratory part 2 are depicted as transition periods, illustrated by the gray areas. It becomes clear that the activity randomization for some participants yields three or fewer transitions (**a**), while for others it yields four transitions (**b**). The analysis of laboratory part 2 including all transition periods is referred to as laboratory part 2a and both, the white and the grey marked periods are analyzed. Laboratory part 2b refers to the same observation protocol with the transition periods excluded from the analysis.

In order to generalize to all individual walking paces in laboratory part 2, participants were instructed to walk according to their own pace. Furthermore, we aimed to test whether an inclusion of all periods including posture transitions (laboratory part 2a), as occurs in daily life tracking, would yield a difference compared to when we excluded transitions (laboratory part 2b). Participants were instructed to perform each of the activities (sitting, standing, walking slowly, rapidly, and jogging) for 2 min before hearing a count-down of 10 s indicating the upcoming activity. Including all transitions, laboratory part 2a produced 10 min of data per participant. Depending on the protocol’s order of postures (e.g., walk rapidly, walk slowly, *transition*, stand, *transition*, sit, *transition*, jog) and, thus, the number of transitions to be excluded, laboratory part 2b revealed 6 to 8 min of data per participant (Figure 3).

##### Laboratory Part 3—Sitting down and Standing up: The Influence of Different Speeds

**Figure 4:**
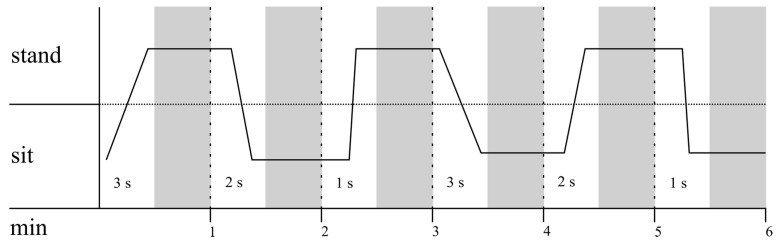
Laboratory part 3: Example of possible activity protocol of the first half of this laboratory part. Process is portrayed as a function of time. Thirty-second periods allocated for the participants’ posture changes in one of the three different speeds (1, 2, and 3 s) were excluded. The validity of sitting and standing after the participants had performed their transitions are depicted by the gray areas.

Since the VitaBit algorithm distinguishes between sitting and standing in case there is no walking detected, laboratory part 3 challenged the device by including slower accelerations. Participants were instructed to take no more than 30 s for their posture changes, which were sitting down or getting up for 1, 2, or 3 s. These transition times were then followed by at least 30 s of standing or sitting, which were the objects of analysis. Each pace of sitting down and getting up was performed twice, resulting in 6 min of data per participant after exclusion of transitions (Figure 4).

#### 2.3.2. Validity of the VitaBit in a Free-Living Condition

For the free-living study, the ActiGraph with a sampling rate of 30 Hz served as a criterion measurement and participants were instructed to wear both the VitaBit and the ActiGraph at the same time. The ActiGraph is a tri-axial MEMS accelerometer and measures 4.6 cm × 3.3 cm × 1.5 cm. With the help of an elastic strap, it can be placed around the wrist, waist, ankle, or thigh [16]. Although the ActiGraph was already found to be a valid detector for sitting, standing, and walking [17,18,19], different inclinometer algorithms, wearing locations, and performance calculations, respectively, make adequate judgment for our purposes difficult. Therefore, a laboratory sub-study was conducted for two purposes: determining reference performance values (e.g., whether the free-living sensitivity of the VitaBit with ActiGraph as the criterion measurement was equal to the laboratory sensitivity) and revealing the eligibility of the ActiGraph as criterion measurement tool. In the laboratory sub-study, participants wore the ActiGraph as well as the VitaBit device and followed the same protocol as in the VitaBit laboratory conditions (see Section 2.3.1).

For the free-living condition, the participants wore the ActiGraph on their thigh [20] while wearing the VitaBit in their trouser pockets or, if no or only loose pockets were available, attached to a garment around the same position, on at least one typical week- or weekend day. Participants were asked to synchronize the VitaBit sensor with their VitaBit application on their phones after they wore the trackers in their daily lives.

### 2.4. Data Analysis

The VitaBit firmware uses a unique algorithm to classify 30-s periods into the categories of sitting, standing, walking and idle data, according to the three-dimensional acceleration data. The researcher exported the raw activity data as .csv files. Each row was dedicated to a certain 30-s period (in UTC time zone) of a user (encrypted as user identifier) indicating the activity as a Boolean variable (e.g., sitting = yes, standing = no, walking = no). The ActiGraph acceleration data were cleaned and converted into activities using the proprietary wearing time validation and the inclinometer function of the ActiGraph software (ActiLife 6.11.9., ActiGraph, Pensacola, FL, USA) [21]. Data were further cleaned and adapted to the observation protocols as well as to the VitaBit output using the “PhysicalActivity” package [22] and general functions in R [23]. In the free-living part, the data from those days were excluded if the participant wore both trackers for less than 5 h per day in order to make adequate device comparisons. Furthermore, if one of the trackers did not register a 30-s period for technical reasons, this period was excluded, hence all analysis are based on merged data. In the laboratory study, 14 datasets of 30 s were not tracked by VitaBit due to late initialization and were excluded. In the free-living study, 3617 of the 33,786 datasets were not tracked by the VitaBit while being tracked by the ActiGraph. Among the data not detected by the VitaBit in the free-living part, 464 datasets at the beginnings and ends of the wearing days are likely due to non-wearing or late initialization of the VitaBit device, and 2895 of the non-tracked datasets were due to wearing only the ActiGraph but not the VitaBit device. Therefore, 258 datasets (0.8%) that were excluded in the free-living part are unexplained lapses of the VitaBit.

Since the ActiGraph displayed the data in second-to-second periods while the VitaBit presented them in 30-s periods, the multiple ActiGraph activities of some 30-s periods needed to be reduced to one single activity. Instead of excluding those ambivalent intervals and, thus, excluding critical transition times, priority was given to the most dominant ActiGraph activity. Therefore, the activity of a 30-s period would constitute the ActiGraph activity performed for the longest time during these 30 s. In case two activities (e.g., sitting, and standing, both for 15 s) or all three activities (e.g., sitting, standing, and walking, all for 10 s) dominated, priority was allocated to sitting, then to standing, and last to walking. For example, 8 s sitting, 11 s standing, and 11 s walking would reveal standing. Those ambivalent situations occurred in 13 out of 1789 (0.73%) periods for the sub-study and in 333 out of 33,785 (0.99%) periods for the free-living periods.

After synchronization of VitaBit with the direct observation protocol data and the ActiGraph data, the performance values, as indicated in Equations (1)–(4), were calculated for each of the three laboratory parts and for each of the three activities on the basis of positives and negatives (Table 1) of all 30-s periods using R [23]. More precisely, sensitivity indicates the percentage of correctly detected activity (e.g., how often, if a person is sitting, this is detected by the device), while specificity refers to the percentage of correctly detected negatives (e.g., how often, if a person is NOT sitting—hence standing or walking—this is detected as non-sitting). Positive (precision) and negative predictive rates indicate the proportion of correctly detected activities (and negatives) among all detected activities. For instance, if the VitaBit displayed the participant as sitting, how much was the participant actually sitting?

Sensitivity = (True Positives)/(True Positives + False Negatives)(1)

Specificity = (True Negatives)/(True Negatives + False Positives)(2)

PPR = (True Positives)/(True Positives + False Positives)(3)

NPR = (True Negatives)/(True Negatives + False Negatives)(4)

**Figure 5:**
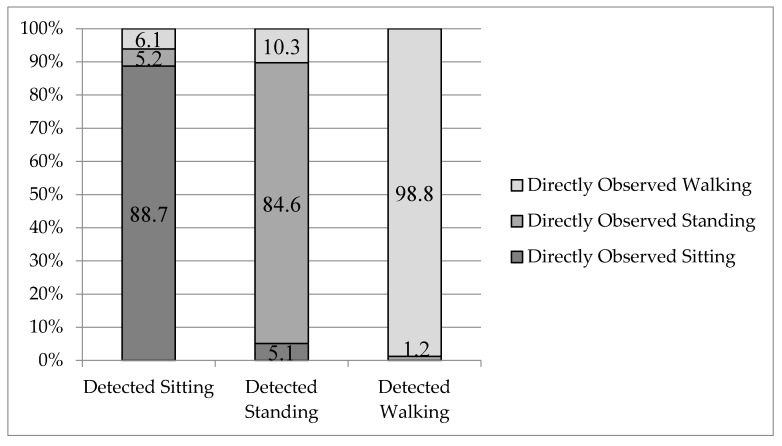
ActiGraph detections and the proportion of actual underlying behaviors.

Since the ActiGraph as a criterion measurement for the free-living part did not reveal perfect validity, a new free-living activity distribution was estimated based on the ActiGraph precision values obtained during the sub-study. For instance, if a person was actually standing but not detected as such by the ActiGraph (*100%* − *PPR_standing_ActiGraph)*, the ActiGraph sometimes detected sitting *(Standing_As_Sitting)* or walking *(Standing_As_Walking)* instead. Among all sitting detection, “Standing_As_Sitting” is, therefore, the part, which is, according to the laboratory sub-study, actually standing. Hence, all sitting detection of the ActiGraph can be divided into correctly detected sitting *(PPR_sitting_ActiGraph)*, in actual standing that was misdetected as sitting, as well as in actual walking that was misdetected as sitting. If we calculate all those actual activity portions for each detected activity based on the sub-study (Figure 5), and multiply the portions by the minutes of detected activities from the free-living part, we get a more likely actual activity distribution (see equations below).

Estimated actual sitting = (PPR_sitting_ActiGraph × Detected_Sitting) + ((100% − PPR_standing_ActiGraph) × Sitting _As_Standing × Detected_Standing) + ((100% − PPR_walking_ActiGraph) × Sitting_As_Walking × Detected_Walking)(5)

Estimated actual standing = (PPR_standing_ActiGraph × Detected_Standing) + ((100% − PPR_sitting_ActiGraph) × Standing _As_Sitting × Detected_Sitting) + ((100% − PPR_walking_ActiGraph) × Standing_As_Walking × Detected_Walking),(6)

Estimated actual walking = (PPR_walking_ActiGraph × Detected_walking) + ((100% − PPR_sitting_ActiGraph) × Walking _As_Sitting × Detected_Sitting) + ((100% − PPR_standing_ActiGraph) × Walking_As_Standing × Detected_Standing).(7)

## 3. Results

### 3.1. Laboratory Study

In the laboratory condition, 11 volunteers (nine females, two males; mean (SD) age 27.1 (5.8) years; height 172.0 (8.9) cm) participated. All participants followed an observation protocol including on average 5.7 (0.9) min sitting (3 min excluded for one participant due to late initialization of VitaBit), 5.5 (1.5) min standing (5 min excluded for one participant due to late initialization of VitaBit), and 10 min walking. The VitaBit detected 6.3 (2.7), 4.9 (2.6), and 10.0 (0.5) min sitting, standing, and walking, respectively (Table 2).

The statistical measurements of performance for all laboratory parts (Table 3) ranged from 75.6% (PPR, laboratory part 2) to 98.1% (NPR, part 1) for sitting, from 70.0% (sensitivity, part 2) to 97.3% (specificity, part 1) for standing, and from 92.9% (specificity, part 2) to 100% (specificity and PPR, part 1) for walking. Transitions periods to and from sitting affected measurements of performance for sitting: When excluding transition periods in laboratory part 2b, sensitivity (+8.4%) and PPR (+7.2%) for sitting were improved the most, while the other activities’ performance values were affected for 2.3% ± 1.4%.

Regarding sensitivity, in 14.3%, 25.4%, and 2.7% the VitaBit device did not successfully detect sitting, standing, and walking, respectively. When it did not successfully detect sitting, 72.2% were detected as standing, 27.8% as walking. When it was supposed to detect standing, 96.8% was detected as sitting and 3.2% as walking. For walking, it measured standing in 66.7% of the cases and idle data in 33.3% of the cases.

### 3.2. Free-Living Condition

#### 3.2.1. Sub-Study

Eleven volunteers were invited and followed the protocol of the laboratory sub-study. After two persons dropped out because the VitaBit was worn on the wrong place or did not successfully synchronize with the app, nine (six females, three males) participants (mean (SD) age 27.2 (7.7), height 170.2 (8.1) cm) were included in the analysis. All participants followed an observation protocol including on average 6 min sitting, 5.9 (0.2) min standing (30 s excluded for one participant due to late initialization of ActiGraph), and 10 min walking, where 6.4 (0.9), 6.5 (1.4), and 9.1 (1.4) min sitting, standing, and walking, respectively, were detected by the ActiGraph (Table 4).

ActiGraph performance in all laboratory sub-study parts (Table 5) ranged from 81.8% (PPR, laboratory part 1) to 100% (sensitivity and NPR, part 1) for sitting, from 73.9% (PPR, part 1) to 99.3% (NPR, part 2) for standing, and from 86.1% (specificity, part 1) to 99% (PPR, part 2) for walking. With sensitivity and specificity values ranging from 86.1% to 100%, it was judged as eligible for the criterion measurement in the free-living condition.

PPR: When the ActiGraph device detected sitting, standing, and walking, respectively, 11.3%, 15.4%, and 1.2% was misclassified. When it measured sitting while a person was not, for 46.2% the person was standing *(Standing_As_Sitting)*, and for 53.8% walking *(Walking_As_Sitting)*. When it misdetected standing, 33.3% was actually sitting *(Sitting_As_Standing)*, and 66.6% was walking *(Walking_As_Standing)*. For wrongly measured walking, the participant was standing *(Standing_As_Walking)* in 100% of the cases (Figure 5). 

#### 3.2.2. Free-Living

In the free-living condition, seven volunteers (six females, one male; mean (SD) age 34 (10), 167 (9) cm) wore the trackers on three (one) days per person. They wore the two devices on average for 774 (232) min per day (ranging from 323 to 1102 min per day). According to the ActiGraph, participants’ daily sitting time was 489 (171) min, standing time 220 (109) min, and walking time was 64 (40) min. Inferring from the PPR values of the sub-study, it is likely that the participants were actually sitting for 445 (153) min, standing for 212 (94) min, and walking for 116 (49) min. The VitaBit detected them sitting for 444 (200) min, standing for 241 (125) min, and walking for 89 (57) min (Table 6).

The activity distributions of estimated activity and VitaBit corresponded: 57.4% (vs. 57.5% estimated) average sitting, 31.1% (vs. 27.5% estimated) average standing and 11.5% (vs. 15.0% estimated) average activity per day. The VitaBit performance with ActiGraph as criterion measurement (Table 7) deviated from the performance values of the sub-study performance values with ActiGraph as reference value (Appendix B). The performance during free-living conditions ranged from 72.6% (NPR; sub-study: 97.9%) to 89.8% (PPR; sub-study: 88.7%) for sitting, from 62.8% (PPR; sub-study: 84.6%) to 87.1% (NPR; sub-study: 97.1%) for standing, and from 47.9% (PPR; sub-study: 98.8%) to 96.8% (NPR; sub-study: 91.8%) for walking. 

## 4. Discussion

Next to PA measurements, objective measurements of SB are essential to counter its detrimental effects, whether as a monitoring tool for the end user, or as a tool to improve interventions and refine recommendations. The major finding of the laboratory study is that the VitaBit is a specific and precise (PPR) tool to distinguish between sitting, standing, and walking modes. Inferring from direct observation, this applies for sitting still, standing, and regular walking. The performance of the VitaBit with the ActiGraph as criterion measurement and calculated on a minute-by-minute base in the free-living condition is low compared to the sub-study. Yet, both trackers show very similar activity distributions. On a day-to-day basis and for normal sitting, standing, and walking, this makes the VitaBit eligible for measuring SB and its antagonist behaviors and it can be used as a low-cost and user-friendly tool for developing, monitoring, and improving SB change programs.

In the laboratory study, it was found that VitaBit was especially sensitive for walking and sitting. For instance, if the user is not sedentary, over 91% is indeed not displayed as sitting, similar to the value of 95.5% of the ActiGraph. Moreover, the distinction between walking and non-activity of over 97% was higher compared to the ActiGraph output. Therefore, the time of walking or other activities can be trusted, while the remainder concerning the distinction between sitting and standing is still questionable. The sensitivity of standing detection showed the lowest performance in the laboratory condition, while the PPR of standing was the lowest value for the ActiGraph according to the sub-study. Nevertheless, the value of over 70% for accurately distinguishing standing still from sitting can be considered high, since other accelerometer studies overcome this issue by considering standing as SB [18,24]. If we consider the activity distribution and not the minute-by-minute comparison, the interpretation of results is more positive: Since the specificity of walking was very high, it is likely that the VitaBit indicates sitting as the remainder of non-detected standing. Similarly, if sitting is not detected it is likely displayed as standing, revealing at least partly compensated daily sitting and standing accumulations. This is in accordance with the very similar activity distributions of the VitaBit and direct observation in the laboratory part.

The daily activity distributions of the VitaBit compared to the ActiGraph and estimated activity distribution deviate minimally. This is in accordance with former validation studies examining other wearable monitors [18,19,21]. Nevertheless, the free-living performance values seem quite low calculated on a minute-to-minute base, while the reference laboratory sub-study of VitaBit performance with ActiGraph as criterion revealed higher performance values (e.g., sensitivity: 71.4% sitting, 71.8% standing, 96.9% walking; Appendix B). It is possible that the low free-living tracker correspondence is due to alternative behaviors such as car driving, cycling, or active sitting and to more transitions between postures, revealing different device outcomes caused by different firmware algorithms or high- and low-pass filters of the VitaBit in comparison to the ActiGraph. Therefore, the results from the laboratory study and the sub-study of (at least one of) the devices are likely not directly transferable to free-living behaviors. 

Despite very high sensitivity values for sitting of other devices of around 99.7% (Activpal) and 95.1% (ActiGraph GT3X), the sensitivity values of 85.7% (laboratory condition) and 81.5% (free-living study) for a low-budget and easy-to-deploy sitting monitor with user-friendly software are satisfactory [19,25]. Consequently, researchers will need fewer resources for getting and cleaning data and might face fewer compliance issues from their study participants. Furthermore, the current study performed a minute-wise comparison and challenged the VitaBit device by including all transitions and direct observation. Besides relatively high performance values, the individual benefits from the VitaBit tool through their entire behavioral change process. This encompasses the (autonomous) monitoring process, short- and long-term goal setting, and overcoming motivational or social hurdles with the help of individualized feedback from a coach or competition with others. Some of these features can be used if a user joins an environment, which can be done anonymously without sharing personal information. Those factors are often summarized as tailoring and user support and can increase program engagement, and therefore behavioral change [26]. 

### Limitations

Since the VitaBit is based on accelerations and smaller people’s thighs cover shorter transition distances when sitting down or getting up, the device’s performance might depend on subject height (results not reported: this statement is based on preliminary findings of a primary performance comparison between shorter and taller subjects). Furthermore, the population on average met the current sitting and standing recommendations [27]. Therefore, replication studies are needed to confirm our results. Although we “challenged” the VitaBit with tight transition times (laboratory part 2), slow sitting–standing transitions (laboratory part 3), and the requirement to distinguish between standing still and sitting, we observed a limited number of activities. For minute-by-minute values, as opposed to activity distribution, the laboratory findings can, therefore, only be applied to daily life activities that are not specific, such as active sitting. We recommend a direct observational free-living study or a laboratory study with a wider activity range [19,24].

## 5. Conclusions

Our findings support the usage of the VitaBit device for research, behavioral change specialists, as well as for the individual who aims for a healthier sit–stand–walk pattern. The VitaBit constitutes a compromise between best-practice, highly sensitive SB trackers currently successfully applied in research and commercially available PA trackers effectively used in PA interventions [11,13,14,15,28].

Since the VitaBit shows high performance on a minute-by-minute basis, the device is a valid tool to detect even slight sedentary interruptions. Therefore, sedentary pattern measures, such as number of sitting bouts or breaks per sedentary hour, can be assessed and, with the help of a combining algorithm, such as the VitaBit score, be validated against health indicators (e.g., glucose or insulin levels). Hence, the current lack of a globally accepted and validated sedentary pattern recommendation could be overcome, enabling tailored suggestions such as “interrupt your sitting every hour for at least 2 min of walking to achieve a significant health boost today.” In accordance, we suggest a future validation study including the step count tool of the VitaBit to investigate putative differences between activity levels when interrupting sitting.

Finally, this study can be used to improve the VitaBit device. One suggestion to improve the sit vs. stand distinction could be to implement a gyroscope in addition to the accelerometer. This would reveal a more stable, absolute system of coordinates as a reference, and produce less acceleration-caused confusion for activities such as car driving or active sitting. Nevertheless, this would increase the device’s power consumption and the producer would again arrive at a trade-off between accuracy and user-engagement factors.

## Figures and Tables

**Table 1:** Confusion matrix for the example of sitting compared to direct observation.

		True Condition	
		Observed Sitting	Observed Non-Sitting (Standing or Walking)
**Prediction condition**	**Detected sitting by VitaBit**	True Positive (TP)	False Positive (FP)
	**Detected non-sitting by VitaBit (standing or walking)**	False Negative (FN)	True Negative (TN)

**Table 2:** Laboratory: Description of population and activity distributions.

Condition	VitaBit		Direct Observation	
(*N* = 11)	Mean ± SD	Range	Mean ± SD	Range
Age (years)	27.1 ± 5.8	22–38		
Height (cm)	172.0 ± 8.9	156–181		
Sitting (min)	6.3 ± 2.7	2–11	5.7 ± 0.9 ^a^	3–6 ^a^
Standing (min)	4.9 ± 2.6	0.5–9.5	5.5 ± 1.5 ^a^	1–6 ^a^
Walking (min)	10.0 ± 0.5	9–10.5	10.0 ± 0	10–10

Sitting, standing and walking data refer to the average activity performed by each participant in the laboratory condition. ^a^ Five standing and three sitting minutes were excluded in one participant as the VitaBit did not initialize.

**Table 3:** Laboratory: performance of the VitaBit with direct observation as criterion measurement.

Laboratory Part	Performance Measurement	Sitting	Standing	Walking
1	Sensitivity ^a^	90.9	77.3	98.9
	Specificity ^b^	95.5	97.3	100
	PPR ^c^	80	85	100
	NPR ^d^	98.1	95.5	97.8
2a ^e^	Sensitivity ^a^	77.3	70	96.2
	Specificity ^b^	93.6	95.5	92.9
	PPR ^c^	75.6	77.8	95.5
	NPR ^d^	94.2	93.3	94
2b ^f^	Sensitivity ^a^	85.7 (+8.4)	72.7 (+2.7)	97.4 (+1.2)
	Specificity ^b^	96.3 (+2.7)	96.5 (+1)	98 (+5.1)
	PPR ^c^	82.8 (+7.2)	76.2 (+1.6)	99.1 (+3.6)
	NPR ^d^	97 (+2.8)	95.8 (+2.5)	94.2 (+0.2)
3	Sensitivity ^a^	90	76.7	-
	Specificity ^b^	76.7	90	100
	PPR ^c^	79.4	88.5	-
	NPR ^d^	88.5	79.4	100
All parts	Sensitivity ^a^	85.7	74.6	97.3
	Specificity ^b^	91.2	95.1	97.6
	PPR ^c^	78.3	84.3	97.3
	NPR ^d^	94.5	91.4	97.6

^a^ ratio of correctly detected activity and observed activity: TP/(TP+FN); ^b^ proportion of correctly detected negatives (activity distinct from the concerning activity) and negatives detected by observation: TN/(TN+FP); ^c^ proportion of TPs within detected activity: TP/(TP+FP); ^d^ proportion of TNs within detected negatives: TN/(TN+FN); ^e^ incl. transition periods; ^f^ excl. transition periods (improvement compared to values from 2a).

**Table 4:** Sub-study: Description of population and activity distributions.

Condition	ActiGraph		Direct Observation	
(*N* = 9)	Mean ± SD	Range	Mean ± SD	Range
Age (years)	27.2 ± 7.7	22–47		
Height (cm)	170.2 ± 8.1	156–182		
Sitting (min)	6.4 ± 0.9	6–8.5	6 ± 0	6–6
Standing (min)	6.5 ± 1.4	5.5–10	5.9 ± 0.2 ^a^	5.5–6 ^a^
Walking (min)	9.1 ± 1.4	6–10	10 ± 0	10–10

Sitting, standing and walking data refer to the average activity performed by each participant in the laboratory sub-study. ^a^ 0.5 standing minutes were excluded in one participant as the ActiGraph did not detect any data.

**Table 5:** Sub-study: performance of the ActiGraph with direct observation as criterion measurement.

Laboratory Part	Performance Measurement	Sitting	Standing	Walking
1	Sensitivity ^a^	100	94.4	86.1
	Specificity ^b^	95.6	93.3	97.2
	PPR ^c^	81.8	73.9	98.4
	NPR ^d^	100	98.8	77.8
2a ^e^	Sensitivity ^a^	97.2	97.2	91.7
	Specificity ^b^	97.9	95.1	98.6
	PPR ^c^	92.1	83.3	99
	NPR ^d^	99.3	99.3	88.8
2b ^f^	Sensitivity ^a^	100 (+2.8)	100 (+2.8)	90.1 (−1.6)
	Specificity ^b^	97.3 (−0.6)	94.7 (−0.4)	100 (+1.4)
	PPR ^c^	88 (−4.1)	76 (−7.3)	100 (+1)
	NPR ^d^	100 (+0.7)	100 (+0.7)	82 (−6.8)
3	Sensitivity ^a^	90.7	88.7	-
	Specificity ^b^	88.7	90.7	100
	PPR ^c^	89.1	90.4	-
	NPR ^d^	90.4	89.1	100
All parts	Sensitivity ^a^	94.4	92.5	89.4
	Specificity ^b^	95.5	93.8	99.1
	PPR ^c^	88.7	84.6	98.8
	NPR ^d^	97.9	97.1	91.8

^a^ ratio of correctly detected activity and observed activity: TP/(TP+FN); ^b^ proportion of correctly detected negatives (activity distinct from the concerning activity) and negatives detected by observation: TN/(TN+FP); ^c^ proportion of TPs within detected activity: TP/(TP+FP); ^d^ proportion of TNs within detected negatives: TN/(TN+FN); ^e^ incl. transition periods; ^f^ excl. transition periods.

**Table 6:** Free Living: Description of population and activity distributions.

Condition	VitaBit		ActiGraph		Estimated Actual Activity	
(*N* = 7)	Mean ± SD	Range	Mean ± SD	Range	Mean ± SD	Range
Age (years)	34 ± 10	25–49				
Height (cm)	167 ± 9	155–181				
Matched wearing time (min)	774 ± 232	323–1102	774 ± 232	323–1102	774 ± 232	323–1102
Sitting (min)	444 ± 200	165–850	489 ± 171	272–912	445 ± 153	243–815
Standing (min)	241 ± 125	44–461	220 ± 109	41–397	212 ± 94	49–359
Walking (min)	89 ± 57	7–235	64 ± 40	11–157	116 ± 49	31–210

Sitting, standing and walking data refer to the average activity performed by each participant per day.

**Table 7:** Free living: performance of the VitaBit with ActiGraph as criterion measurement.

Performance Measurement	Sitting	Standing	Walking
Sensitivity ^a^	81.5	68.7	66.0
Specificity ^b^	84.0	83.9	93.5
PPR ^c^	89.8	62.8	47.9
NPR ^d^	72.6	87.1	96.8

^a^ ratio of correctly through the device detected activity and activity from observation protocol: TP/(TP+FN); ^b^ proportion of correctly through the device detected negatives (activity distinct from the concerning activity; does not necessarily need to assess the same activity than being observed) and all negatives detected by observation: TN/(TN+FP); ^c^ proportion of TPs within detected activity: TP/(TP+FP); ^d^ proportion of TNs within detected negatives: TN/(TN+FN).

## Supplementary Materials

The cleaned data and the performance calculations as R-source code are available online at: http://hdl.handle.net/10411/H5PZCT.

## Appendix A. PAR-Q (Physical Activity Readiness Questionnaire)

Please read the questions carefully and answer each one honestly: check YES or NO.

**Table:**

	YES	NO
1. Has your doctor ever said that you have a heart condition and that you should only do physical activity recommended by a doctor?		
2. Do you feel pain in your chest when you do physical activity?		
3. In the past month, have you had chest pain when you were not doing physical activity?		
4. Do you lose your balance because of dizziness or do you ever lose consciousness?		
5. Do you have a bone or joint problem (for example, back, knee or hip) that could be made worse by a change in your physical activity?		
6. Is your doctor currently prescribing drugs (for example, water pills) for your blood pressure or heart condition?		
7. Do you know of any other reason why you should not do physical activity?		

If you have answered “Yes” to one or more of the above questions, consult your physician before engaging in physical activity. Tell your physician which questions you answered “Yes” to. After a medical evaluation, seek advice from your physician on what type of activity is suitable for your current condition.

## Appendix B. Sub-Study Comparing ActiGraph and VitaBit

**Table A1:** Sub-study: Description of population and activity distributions.

Condition	VitaBit		ActiGraph	
(*N* = 7)	Mean ± SD	Range	Mean ± SD	Range
Age (years)	24.9 ± 2.5	22–29		
Height (cm)	168.6 ± 7.9	156–181		
Sitting (min)	6.1 ± 2.1	2–8.5	6.5 ± 1.0	6–8.5
Standing (min)	5.6 ± 2.0	3–9.5	6.1 ± 0.4	5.5–7
Walking (min)	10 ± 0.4	9.5–10.5	9.4 ± 0.9	7.5–10

Activity values based on most accurate values per situation. Observations for laboratory data and ActiGraph measurements for free-living data. Sitting, standing, and walking data refer to the average activity performed by each participant per day.

**Table A2:** Sub-study: performance of the VitaBit with ActiGraph as criterion measurement.

Laboratory Part	Performance Measurement	Sitting	Standing	Walking
1	Sensitivity ^a^	66.7	80	98
	Specificity ^b^	98.5	95.7	81.8
	PPR ^c^	92.3	80	89.3
	NPR ^d^	91.5	95.7	96.4
2a ^e^	Sensitivity ^a^	66.7	66.7	96.2
	Specificity ^b^	91.8	95.5	88.3
	PPR ^c^	69	80	91.7
	NPR ^d^	91	91.3	94.6
2b ^f^	Sensitivity ^a^	70	64.7	98.5
	Specificity ^b^	95.2	96.6	89.2
	PPR ^c^	77.8	78.6	94.3
	NPR ^d^	93	93.3	97.1
3	Sensitivity ^a^	76.7	72.5	-
	Specificity ^b^	72.5	76.7	100
	PPR ^c^	75	74.4	-
	NPR ^d^	74.4	75	100
All parts	Sensitivity ^a^	71.4	71.8	96.9
	Specificity ^b^	90.3	91.9	92.6
	PPR ^c^	75.6	77.2	90.7
	NPR ^d^	88.2	89.5	97.6

^a^ ratio of correctly through the device detected activity and activity from observation protocol: TP/(TP+FN); ^b^ proportion of correctly through the device detected negatives (activity distinct from the concerning activity; does not necessarily need to assess the same activity than being observed) and all negatives detected by observation: TN/(TN+FP); ^c^ proportion of TPs within detected activity: TP/(TP+FP); ^d^ proportion of TNs within detected negatives: TN/(TN+FN); ^e^ including transition periods; ^f^ excluding transition periods.
